# The Rise of Fentanyl: Molecular Aspects and Forensic Investigations

**DOI:** 10.3390/ijms26020444

**Published:** 2025-01-07

**Authors:** Cecilia Barletta, Virginia Di Natale, Massimiliano Esposito, Mario Chisari, Giuseppe Cocimano, Lucio Di Mauro, Monica Salerno, Francesco Sessa

**Affiliations:** 1 Legal Medicine, Department of Medical, Surgical and Advanced Technologies “G.F. Ingrassia”, University of Catania, 95123 Catania, Italy; cecilia.barletta96@gmail.com (C.B.); virginiadinatale18@gmail.com (V.D.N.); dr.luciodimauro@gmail.com (L.D.M.); monica.salerno@unict.it (M.S.); 2 Faculty of Medicine and Surgery, “Kore” University of Enna, 94100 Enna, Italy; massimiliano.esposito@unikore.it; 3 “Rodolico-San Marco” Hospital, Santa Sofia Street, 87, 95121 Catania, Italy; m.chisari@policlinico.unict.it; 4 Department of Mental and Physical Health and Preventive Medicine, University of Campania “Vanvitelli”, 80121 Napoli, Italy; giuseppe.cocimano@unicampania.it

**Keywords:** fentanyl, abuse, synthetic opioid, toxicology, pharmacogenetic

## Abstract

Fentanyl is a synthetic opioid widely used for its potent analgesic effects in chronic pain management and intraoperative anesthesia. However, its high potency, low cost, and accessibility have also made it a significant drug of abuse, contributing to the global opioid epidemic. This review aims to provide an in-depth analysis of fentanyl’s medical applications, pharmacokinetics, metabolism, and pharmacogenetics while examining its adverse effects and forensic implications. Special attention is given to its misuse, polydrug interactions, and the challenges in determining the cause of death in fentanyl-related fatalities. Fentanyl misuse has escalated dramatically, driven by its substitution for heroin and its availability through online platforms, including the dark web. Polydrug use, where fentanyl is combined with substances like xylazine, alcohol, benzodiazepines, or cocaine, exacerbates its toxicity and increases the risk of fatal outcomes. Fentanyl undergoes rapid distribution, metabolism by *CYP3A4* into inactive metabolites, and renal excretion. Genetic polymorphisms in *CYP3A4*, *OPRM1*, and *ABCB1* significantly influence individual responses to fentanyl, affecting its efficacy and potential for toxicity. Fentanyl’s side effects include respiratory depression, cardiac arrhythmias, gastrointestinal dysfunction, and neurocognitive impairments. Chronic misuse disrupts brain function, contributes to mental health disorders, and poses risks for younger and older populations alike. Fentanyl-related deaths require comprehensive forensic investigations, including judicial inspections, autopsies, and toxicological analyses. Additionally, the co-administration of xylazine presents distinct challenges for the scientific community. Histological and immunohistochemical studies are essential for understanding organ-specific damage, while pharmacogenetic testing can identify individual susceptibilities. The growing prevalence of fentanyl abuse highlights the need for robust forensic protocols, advanced research into its pharmacogenetic variability, and strategies to mitigate its misuse. International collaboration, public education, and harm reduction measures are critical for addressing the fentanyl crisis effectively.

## 1. Introduction

Fentanyl, a potent synthetic opioid and μ-opioid receptor agonist, was first synthesized in Belgium in 1960 by Dr. Paul Janssen at Janssen Pharmaceutica [1]. Initially introduced as an intravenous analgesic in 1963 in Europe and later in 1968 in the United States, it has become one of the most widely used opioids worldwide [2,3].

Fentanyl’s exceptional potency—approximately 100 to 200 times greater than morphine—combined with its rapid onset and short duration of action has made it indispensable in medical settings, particularly for managing severe chronic pain and intraoperative anesthesia [4,5,6,7].

The pharmacokinetic properties of fentanyl, including its high lipophilicity and protein-binding characteristics, have facilitated the development of diverse administration routes. These include intravenous injections, transmucosal and sublingual formulations, nasal sprays, and transdermal patches [8,9,10,11]. While these attributes enhance its clinical utility, they also contribute to its high potential for misuse. The methods of illicit consumption range from injecting extracted fentanyl to inhaling vapors from heated patches, practices that significantly increase overdose risks [12,13].

In recent decades, the misuse of fentanyl has reached epidemic proportions, driven by its availability on the dark web, low cost, and frequent adulteration with other drugs, such as xylazine, often without the user’s knowledge [14,15]. This has resulted in a sharp rise in overdose deaths globally, with the United States and parts of Europe experiencing particularly severe impacts [16].

This review explores fentanyl’s pharmacokinetics, metabolism, and pharmacogenetics, highlighting its adverse effects, forensic challenges, and the complexities surrounding its misuse. A special focus is placed on its forensic implications, including post-mortem investigations, histopathological findings, and toxicological analyses. The aim is to provide a comprehensive understanding of fentanyl’s dual role as both a lifesaving medication and a significant contributor to the opioid epidemic.

## 2. Fentanyl Abuse

The rise in fentanyl-related deaths underscores a growing opioid epidemic, with increasing abuse of fentanyl and its analogs often occurring alongside heroin use [17,18]. Alarming epidemiological and forensic reports, particularly from the last two decades, indicate a significant rise in the illicit use of fentanyl, especially in North America and Europe [19,20]. Toxicological evidence shows that fentanyl is frequently used in polydrug combinations, often mixed with substances like heroin to enhance effects and reduce costs [21,22]. Of particular concern is the co-administration of fentanyl and xylazine, which has emerged as a significant public health issue due to its synergistic lethality. This combination markedly increases the toxic effects of each drug, resulting in rapid and severe outcomes [23].

Abuse, defined as the excessive, illegitimate, or inappropriate use of a substance, often for psychotropic effects, differs from misuse, which refers to the use of a substance in a manner that does not adhere to medical indications or prescribed dosing [24]. In the medical setting, misuse by anesthesiologists and surgeons was documented as early as the 1970s. This misuse was often unintentional and was facilitated by the drug’s easy availability in hospitals. Fentanyl and sufentanil were among the most misused opioids by healthcare professionals [25,26]. However, the dramatic increase in fentanyl-related deaths today is primarily connected to its illicit use [27].

Fentanyl can be administered through various methods (see Table 1), with one of the most common involving the extraction of the drug from transdermal patches [28]. The extracted fentanyl is then consumed in various ways, such as injection into central venous catheters, inhalation, rectal administration, or boiling for oral ingestion [29,30]. These methods have facilitated widespread abuse, underscoring the urgent need for stricter control and enhanced monitoring.

### 2.1. Increasing Trends: Online Markets and the Dark Web

Several factors contribute to the rising misuse of fentanyl, with one significant driver being its substitution for heroin. Fentanyl’s greater potency, easier availability, and lower legal penalties compared to heroin make it particularly appealing to illicit users [31,32].

Online platforms, particularly the “dark web”, have become key hubs for purchasing illegal drugs anonymously [33]. The dark web, a collection of encrypted websites, protects user identities through anonymization tools that obscure their activities [34]. These platforms, often referred to as darknet markets or cryptomarkets, facilitate the sale and distribution of fentanyl and its analogs while maintaining user anonymity through advanced encryption techniques [35].

Platforms like Darktrend, a semi-automated system developed to monitor advertisements for fentanyl and other opioids, have tracked the prevalence of these substances [36]. Notable cryptomarkets include Agora, shut down by the FBI in 2015, and Dream Market, which hosted over 60,000 drug-related ads before its closure. These online spaces have played a significant role in increasing fentanyl’s availability, contributing to its widespread abuse [37,38].

### 2.2. Combination with Xylazine

Xylazine, a non-opioid veterinary sedative primarily used for large animals, has emerged as a concerning adulterant in the illicit drug supply, particularly when combined with fentanyl. Known colloquially as “tranq” or “tranq dope”, the mixture of xylazine and fentanyl poses significant risks to users, amplifying the already severe health consequences associated with fentanyl abuse. This combination has become increasingly prevalent in the United States and other regions, complicating public health efforts to address the opioid epidemic [39,40]. Alarmingly, xylazine has proliferated in the unregulated drug market, frequently found adulterating the fentanyl supply. Individuals seeking these substances may turn to the black market or dark web, where such drugs are readily available due to their illicit nature [41].

The motivation to combine fentanyl with xylazine lies primarily in its ability to enhance and prolong the effects of fentanyl while reducing the production costs for dealers. Xylazine, a non-opioid sedative, intensifies fentanyl’s sedative and analgesic effects, creating a longer-lasting high for users. This combination is appealing to individuals seeking more potent or sustained experiences. For dealers, xylazine is inexpensive, widely accessible as a veterinary drug, and not yet classified as a controlled substance in many jurisdictions, making it an ideal adulterant to stretch fentanyl supplies [42].

When combined, these substances create a unique pharmacological synergy: fentanyl’s rapid onset and potent analgesia are compounded by xylazine’s profound sedation. This combination often leaves users in an immobilized, semi-conscious state that has earned the nickname “zombie-like effect”. However, this practice significantly increases the risk of fatal overdoses, as xylazine does not respond to naloxone (a lifesaving opioid antagonist) and exacerbates respiratory depression and cardiovascular effects [43,44,45,46,47].

### 2.3. Combination with Other Drugs

Polydrug use involving fentanyl is a well-documented phenomenon. Commonly co-used substances include nicotine, cannabis, and cocaine, which can exacerbate fentanyl’s effects and risks. Fentanyl is pharmacologically potent, requiring only small amounts to produce effects, but its narrow therapeutic window makes it highly dangerous [48,49,50,51,52].

Certain drugs can significantly alter fentanyl’s pharmacokinetics. For instance, ritonavir and calcium channel blockers like diltiazem inhibit CYP3A4 (cytochrome P450 3A4) metabolism, increasing plasma fentanyl levels and prolonging its effects. Conversely, fentanyl can inhibit the clearance of sedative drugs, such as midazolam, compounding the risk of respiratory depression [4].

The combination of fentanyl with cocaine or alcohol has been linked to acute and chronic myocardial damage. Cocaine exacerbates cardiovascular stress by inducing peripheral vasoconstriction, increasing endothelin levels, and disrupting intracellular calcium regulation [53,54,55,56]. These combined effects significantly heighten the risks of arrhythmias, ischemia, myocardial infarction, and sudden cardiac death [57,58].

Alarmingly, since 2013, illicitly manufactured fentanyl and its analogs have increasingly appeared on the streets, often mixed with or sold as heroin. Many users remain unaware of these adulterations, dramatically increasing the risk of overdose and fatal outcomes [59,60,61].

## 3. Pharmacokinetics, Metabolism, and Pharmacogenetics of Fentanyl

The pharmacokinetics of fentanyl involve rapid distribution, metabolism, and elimination, influenced by its high lipophilicity and protein-binding characteristics [62]. Once administered, fentanyl is quickly distributed to highly perfused tissues such as the brain, lungs, and heart due to its lipid solubility [63]. It later accumulates in less vascularized tissues, including muscles and adipose tissue, which act as a drug reservoir, contributing to prolonged effects even after administration ceases [62,63].

Fentanyl’s plasma protein binding, primarily to albumin and α1-acid glycoprotein, is influenced by factors such as plasma pH and protein levels. Changes in these parameters significantly affect the drug’s free concentration and bioavailability, potentially altering its therapeutic effects and risks of toxicity [62,64].

Fentanyl is primarily metabolized in the liver and gastrointestinal mucosa by cytochrome P450 enzymes, specifically *CYP3A4*, into norfentanyl, its major inactive metabolite. This biotransformation predominantly involves N-dealkylation of the piperidine ring. Other metabolic pathways, including hydroxylation and hydrolysis, contribute minimally to the metabolism process and are less clinically relevant [62]. However, impairment in these organs can lead to drug accumulation, increasing the risk of toxicity.

Renal excretion is the primary elimination pathway for fentanyl and its metabolites, with small amounts excreted via the gastrointestinal tract. In individuals with normal renal and hepatic function, fentanyl clearance is typically efficient. However, renal or hepatic impairment can significantly affect fentanyl metabolism and accumulation, potentially leading to toxicity [62]. The fentanyl metabolism may be influenced by the xylazine combination; the xylazine is an α2-adrenergic agonist and may cause central nervous system depression, respiratory depression, bradycardia, and hypotension in humans [65]. Its relatively short half-life (25–30 min) suggests its detection in post-mortem toxicology, indicates its involvement in the final stages of fatal drug use [66]. Moreover, research suggests that xylazine’s pharmacological effects, particularly its α2-adrenergic agonism, may influence the regulation of extracellular matrix components, including collagen synthesis and degradation [67]. This interaction could partially explain the severe tissue necrosis and chronic, non-healing wounds frequently observed in individuals who misuse xylazine. By impairing normal collagen turnover, xylazine may disrupt wound healing processes, leading to fibrotic changes and structural abnormalities in affected tissues [68]. Further investigation into this molecular pathway could provide valuable insights into xylazine’s role in tissue damage and potential therapeutic targets for mitigating its harmful effects.

Fentanyl’s pharmacokinetics and pharmacodynamics are significantly influenced by genetic factors, which contribute to individual variability in drug response [69,70]. Pharmacogenetics provides critical insights into how variations in drug-metabolizing enzymes, transport proteins, and opioid receptors affect fentanyl’s metabolism, efficacy, and toxicity [71,72].

*CYP3A4* and *CYP3A5* are the primary enzymes responsible for fentanyl metabolism [70,73]. Variants in these genes can alter the rate of clearance, with reduced-function variants slowing metabolism and increasing plasma concentrations, thereby heightening the risk of overdose. Conversely, enhanced-function variants may accelerate clearance, necessitating higher doses to achieve therapeutic effects [74,75,76].

Fentanyl’s binding affinities to the different opioid receptors—μ (MOR), κ (KOR), and δ (DOR)—are driven by molecular differences in receptor structure and ligand–receptor interactions. Fentanyl exhibits the highest affinity for MOR, with a Ki in the low nanomolar range (~1.2–1.4 nM). In contrast, fentanyl’s affinity for κ- and δ-opioid receptors is lower, with Ki values of approximately 255 nM and over 1000 nM, respectively. This selectivity profile explains the predominance of MOR-mediated effects, such as analgesia, euphoria, and severe respiratory depression [77,78]. The MOR, encoded by the *OPRM1* (Opioid Receptor Mu 1) gene, mediates fentanyl’s analgesic and adverse effects. A key polymorphism, A118G, modifies receptor binding affinity and sensitivity. Individuals with the GG genotype exhibit reduced receptor sensitivity, requiring higher doses for effective pain relief, while those with other genotypes may be more susceptible to adverse effects due to heightened receptor sensitivity [79,80].

Transport proteins also play a role in fentanyl’s pharmacokinetics. The *ABCB1* (ATP Binding Cassette Subfamily B Member 1) gene encodes P-glycoprotein, a transporter that regulates fentanyl’s passage across the blood–brain barrier. Variants such as C3435T affect transporter activity, influencing fentanyl’s central nervous system concentrations and, consequently, its efficacy and risk for side effects [81,82].

Emerging research highlights the role of other genes, such as *COMT* (Catechol-o-methyltransferase), in fentanyl’s effects. The Val158Met polymorphism in *COMT* affects dopamine metabolism, with the Met/Met genotype linked to enhanced analgesia due to higher dopamine levels in the brain. Other genes, including *UGT2B7* (UDP-Glucuronosyltransferase-2B7), *CGRP* (Calcitonin gene-related peptide), and *CYP2D6* (Cytochrome P450 2D6), contribute to individual variability in fentanyl metabolism and response, though their specific roles are less well-defined [83].

These genetic factors, combined with fentanyl’s unique pharmacokinetics, underscore the complexity of optimizing its use in clinical settings and managing the risks associated with its potency and potential for misuse [84]. In this way, this in silico study could be very helpful in the prediction of pharmacogenetics effects [85].

## 4. Fentanyl’s Adverse Effects

Fentanyl, along with other synthetic opioids such as methadone and oxycodone, exhibits stronger yet shorter-lasting effects compared to morphine, making it highly suitable for intraoperative anesthesia and chronic pain management. Its exceptional analgesic potency enables effective pain relief even at low doses [86].

Fentanyl’s binding affinity to μ-, κ-, and δ-opioid receptors not only drives its analgesic properties but also accounts for its extensive side effect profile. These range from mild, such as itching and nausea, to severe and life-threatening, such as respiratory depression and cardiac arrest [13,87,88]. Psychostimulant side effects include analgesia, itching, and euphoria, while central nervous system effects may include delirium, sedation, nausea, vomiting, and constipation [89]. Dizziness and respiratory depression are notable concerns, with the latter potentially leading to apnea and death if dosages exceed safe limits. Fentanyl-induced respiratory depression is exacerbated by its vagus nerve stimulation effects, which can also cause bradycardia. At higher doses, fentanyl can cause loss of consciousness and anesthesia, irrespective of the route of administration [90].

The risks associated with fentanyl increase substantially when combined with other CNS (central nervous system) depressants, such as alcohol or benzodiazepines [86]. These combinations significantly increase the likelihood of severe adverse effects, including respiratory depression, fatal cardiac arrhythmias, and profound sedation. Unlike some other opioids, fentanyl can trigger these outcomes without elevating plasma histamine levels, which makes it distinct [91]. Moreover, as previously described, there is a growing threat to the combined use of xylazine and fentanyl. Particularly, the chronic use of xylazine is associated with additional severe health complications, such as chronic skin ulcers and necrosis, cardiovascular and respiratory compromission, and withdrawal symptoms. Skin damage is related to the vasoconstrictive properties of xylazine that impair blood flow, particularly in peripheral tissues, leading to necrosis, ulcerations, and non-healing wounds. These wounds are often severe, exposing underlying tissues and becoming easily infected. In many cases, amputation becomes the only viable treatment option [68,92,93,94].

**Figure 1:**
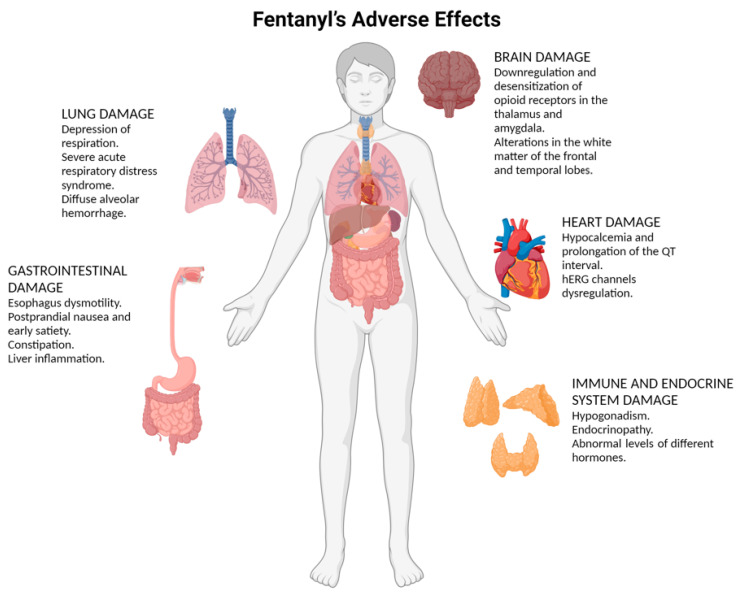
This figure provides an overview of the complex and far-reaching adverse effects fentanyl has on various bodily systems. (created with BioRender, https://biorender.com).

The main adverse effects related to fentanyl abuse are summarized in Figure 1.

Elderly patients are particularly susceptible to fentanyl side effects. Commonly reported symptoms include vertigo, migraines, confusion, dizziness, and hallucinations, which not only reduce quality of life but also increase the risk of falls and associated complications that in elderly patients are associated with an increased death risk [95,96]. In younger individuals, fentanyl’s high potency and rapid onset make it especially hazardous. A notable adverse effect is chest wall rigidity, a condition impairing ventilation and chest movement, which can lead to respiratory failure. Chronic misuse in this population may result in neurocognitive deficits, including disruptions in memory, learning, and emotional regulation, alongside increased anxiety, depression, and risky behaviors [97,98].

Individual responses to fentanyl vary widely and are influenced by factors such as age, sex, body weight, pharmacokinetics, comorbidities, polydrug use, and environmental conditions. For instance, younger individuals may experience more pronounced psychostimulant effects, while older adults are more prone to cognitive disturbances. Additionally, underlying health conditions, such as respiratory or cardiovascular disorders, amplify the risks of adverse outcomes [99,100].

Recognizing and understanding these side effects is critical for clinicians prescribing fentanyl, as well as for public health strategies aimed at reducing its misuse. Monitoring patient-specific factors, tailoring doses to individual needs, and identifying early signs of adverse reactions are essential to minimizing risks. In cases of illicit use, the unpredictable purity and potency of fentanyl further heighten the dangers, emphasizing the need for widespread education, harm reduction strategies, and accessible treatment for opioid addiction [101].

### 4.1. Brain Damage

Fentanyl’s high lipophilicity allows it to cross the blood–brain barrier efficiently, where it acts as a potent central nervous system depressant [4,102,103]. By acting on MOR in the periaqueductal gray matter, it inhibits the neurotransmitter gamma-aminobutyric acid (GABA) release and activates potassium channels, modulating the pain pathway originating in the spinal cord [4,102,103]. This mechanism makes fentanyl highly effective for managing chronic pain.

Chronic exposure to fentanyl results in the downregulation and desensitization of opioid receptors in key brain regions, such as the thalamus and amygdala. Over time, these changes contribute to structural and functional alterations in the white matter of the frontal and temporal lobes, resulting in cognitive and emotional impairments [104,105].

A hallmark of opioid overdose is respiratory depression, which, if prolonged, can lead to cerebral hypoxia, resulting in brain damage and neuropsychological deficits [106]. In acute cases, fentanyl alters pain perception, induces euphoria, and promotes relaxation; however, its sedative effects also impact the sleep–wake cycle [107]. Moreover, as previously described, the veterinary sedative xylazine is used as an additive to fentanyl, contributing to the “zombie effect”, characterized by slow movements, a bent posture, and a vacant stare observed in abusers [108]. Furthermore, repeated use of xylazine and fentanyl contributes to significant neurotoxicity, leading to cognitive impairments, memory deficits, and mood disorders. Chronic sedation and hypoxia caused by the combination exacerbate these effects, potentially causing long-term brain damage [39,42,66].

### 4.2. Lung Damage

Fentanyl significantly impacts pulmonary function. Acutely, it suppresses the cough reflex by targeting the medulla oblongata’s cough regulation center and impairing the brainstem’s respiratory control. Additionally, fentanyl acts on chemoreceptors, reducing the body’s respiratory response. This leads to hypoventilation, characterized by irregular, shallow, and slow breathing, which may progress to hypercapnia, hypoxemia, chest wall rigidity, and apnea [109]. These effects are significant contributors to fentanyl-induced fatalities, especially in cases of overdose. Usually, fentanyl overdose can be related to acute lung injury as severe acute respiratory distress syndrome [110]. The administration way could influence lung damage: as recently reported, inhaled fentanyl leads to diffuse alveolar hemorrhage [111]. Chronic administration can induce pulmonary diseases, including interstitial inflammation, fibrosis, bronchospasm, pulmonary edema, and pleural effusions [112].

### 4.3. Heart Damage

Chronic illicit drug use, particularly opioids like fentanyl, poses a significant long-term risk for cardiac dysfunction, including myocardial fibrosis. Morphometric analyses have shown a strong correlation between drug-related damage (DRD) and structural heart changes [113,114]. Recent studies indicate that fentanyl interacts with the hERG (human Ether-a-go-go-Related) channels, which regulate the potassium currents critical for cardiac repolarization [115]. Dysregulation of these channels can lead to hypocalcemia and prolongation of the QT interval, increasing the risk of arrhythmias, torsades de pointes, and sudden cardiac death [116]. These findings underscore the need for caution when administering fentanyl, particularly in individuals with pre-existing cardiac conditions.

Finally, the co-administration of xylazine should be considered: indeed, it induces profound bradycardia and hypotension, thereby exacerbating cardiovascular adverse effects, particularly increasing the risk of fatal arrhythmias and ischemic events [39,42,44,45].

### 4.4. Gastrointestinal Damage

Like other opioids, fentanyl significantly impacts gastrointestinal function. By targeting MOR in the enteric nervous system, it inhibits acetylcholine release, disrupting peristalsis and causing severe constipation. In the esophagus, opioids can cause dysmotility, presenting symptoms like primary achalasia, including dysphagia. In the stomach, they can delay gastric emptying, leading to postprandial nausea and early satiety. In the colon, opioid-induced constipation (OIC) frequently occurs, and a condition known as narcotic bowel syndrome manifests as chronic abdominal pain, nausea, and vomiting without an identifiable cause [117]. Chronic use has been linked to liver damage, with histopathological studies showing hepatocyte necrosis and portal inflammation [118]. Less well-characterized are the potential direct hepatotoxic effects of fentanyl [119]. In overdose cases, elevated IL-6 levels and reduced CYP3A11 (Cytochrome P450 3A11) expression highlight an inflammatory response and impaired hepatic drug metabolism [120]. Interestingly, emerging research has begun exploring the connection between the gut microbiome and drug addiction. Notably, studies indicate that reducing the diversity of the gut microbiome or impairing its normal composition significantly enhances the motivation to seek fentanyl, suggesting a potential role for gut health in modulating addictive behaviors [121].

### 4.5. Immune and Endocrine System Damage

Fentanyl also affects the immune and endocrine systems [122]. Fentanyl abuse, like alcohol, opioids, and anabolic-androgenic steroids, can reduce testosterone production in males by disrupting testicular or hypothalamic-pituitary function [123]. Acute use is associated with reduced plasma levels of cortisol, testosterone, and gonadotropins, while chronic opioid therapy can lead to endocrinopathies in both men and women. These hormonal disruptions compromise immune function, reducing the body’s ability to fight infections and maintain metabolic balance [124]. Although these findings suggest immunosuppressive effects, limitations related to methodological variability, differing opioid dosages, and small sample sizes caution against generalizing immunosuppression as a universal side effect of all opioids [125].

Finally, considering the combined use of xylazine, chronic exposure to this drug has been linked to weakened immune responses [126]. This immunosuppression, coupled with poor wound healing and unsanitary injection practices, increases vulnerability to severe bacterial infections, including cellulitis, abscesses, and sepsis.

## 5. Post-Mortem Investigation in Fentanyl-Related Deaths

### 5.1. Crime Scene Investigation

A thorough crime scene investigation is critical for establishing the context of fentanyl-related deaths. Investigators should document and collect evidence such as syringes, needles, transdermal patches, spoons, aluminum foil, and other paraphernalia associated with fentanyl use. Items such as discarded packaging or solvent containers may indicate illicit extraction methods [127,128]. The presence of prescription fentanyl, such as patches or vials, should be compared with medical records to determine whether misuse or diversion occurred. Accurate documentation of the scene, including the positioning of the body and nearby materials, is essential for reconstructing the events leading to death [129]. Fentanyl poses significant risks to first responders and investigators, as even small quantities can be fatal; therefore, the use of personal protective equipment (PPE) is essential, and rapid administration of naloxone, often in repeated doses due to its short duration of action, can be lifesaving in such situations [130].

### 5.2. External Examination

During the external examination, the forensic pathologist should carefully inspect the body for signs indicative of fentanyl misuse or overdose. These may include needle punctures, particularly in less visible areas like the feet or groin, and signs of venipuncture. Pupillary constriction (miosis), a hallmark of opioid toxicity, and frothy material (“mushroom plume”) around the mouth or nose suggest respiratory distress or pulmonary edema. Discoloration of the skin, such as cyanosis or purple areas, may indicate hypoxia prior to death. Additionally, the presence of any rashes or irritation near transdermal patch sites should be noted [131,132].

### 5.3. Autopsy Investigation

At autopsy, a systematic approach should be taken to examine each organ, including weighing, measuring, and performing a detailed macroscopic inspection. Observations such as the presence of petechiae in the lungs or pleura, organ congestion, or focal hemorrhages should be documented [133,134,135]. Considering fentanyl’s adverse effects, key steps include:

- Brain: Evaluate for cerebral edema, focal hemorrhages, or signs of hypoxic-ischemic injury. Weigh and preserve the brain for histological analysis, particularly the hippocampus and cerebellum.
- Lungs: Note pulmonary edema, frothy fluid in airways, or signs of aspiration pneumonia. Weigh both lungs and assess for amorphous material within the alveoli.
- Heart: Examine for myocardial fibrosis, valvular abnormalities, or signs of arrhythmias such as focal ischemic changes. Document the weight and macroscopic findings.
- Liver: Assess for hepatomegaly, discoloration, or nodularity, indicating chronic damage. Note any portal inflammation or necrosis.
- Gastrointestinal Tract: Inspect for irritation, ulcers, or other lesions associated with opioid use.
- Gonads: In males, examine the testes for reduced size or changes in tubular structure; in females, assess the ovaries for signs of chronic endocrine disruption.

After the initial gross examination, all organs should be fixed in formalin for subsequent histological, immunohistochemical, toxicological, and genetic analyses. Samples of blood (preferably from the femoral vein), urine, vitreous humor, gastric contents, and solid tissues (e.g., brain, lungs, liver) should be collected promptly to minimize the post-mortem changes and redistribution effects. Hair samples from the nape or pubic area can provide a long-term drug exposure history [136].

### 5.4. Histological Findings in Fentanyl-Related Deaths

The use of fentanyl can cause a range of toxic effects in various organs, each with distinct histopathological characteristics [137,138]. Histological analysis reveals critical evidence of fentanyl’s effects on the following target organs:

- Brain: Chronic exposure leads to the downregulation of opioid receptors and structural changes in the white matter of the frontal and temporal lobes, resulting in cognitive and emotional impairments. Histologically, hypoxic damage is common, with loss of eosinophilic Purkinje cells in the hippocampus and cerebellum. Neuronal apoptosis, microglial activation, and cortical degeneration are also observed [138,139].
- Lungs: Pulmonary findings include edema, evidenced by a “mushroom plume” of frothy material in the airways and mouth and amorphous eosinophilic deposits in alveolar spaces. Aspiration pneumonia, intra-alveolar hemorrhage, and neutrophilic inflammation are common in cases with a prolonged interval between unconsciousness and death [140].
- Heart: Fentanyl impacts the hERG channel, disrupting potassium currents and prolonging the QT interval, increasing the risk of arrhythmias and sudden cardiac death. Chronic intravenous use may also result in endocarditis and myocardial fibrosis [141].
- Liver: Histopathological changes include hepatocyte necrosis, lymphocyte infiltration, and portal inflammation. Chronic use often coincides with viral hepatitis, particularly hepatitis C, in intravenous drug users [142].
- Gonads: Chronic opioid exposure leads to endocrine disruptions, such as hypogonadism and reduced libido. Histological findings include diminished germ cell maturation and reduced tubular diameter in the testes [123,143].

In Table 2, we summarized the organ, relative toxicity, and histopathology damage.

### 5.5. Immunohistochemical Markers in Fentanyl-Related Deaths

Immunohistochemical markers are valuable for understanding the adverse effects of fentanyl abuse and identifying the underlying mechanisms. These markers can be divided into specific markers, which directly highlight the damage caused by fentanyl, and aspecific markers, which reflect generalized responses such as inflammation or tissue injury [4]. The relevant markers are reported in Table 3.

In the brain, fentanyl’s action can lead to neuronal apoptosis, microglial activation, and white matter degeneration. These effects are often observed in regions like the hippocampus and cerebellum [138,144,145]. A specific marker such as GFAP can be used to identify astrocytic gliosis, a hallmark of neuroinflammation and tissue damage [146]. Additionally, NeuN can be used to assess neuronal viability, and cleaved caspase-3 serves as a marker for apoptosis, indicating neuronal loss [138,147,148,149]. To evaluate microglial activation and neuroinflammation, aspecific markers such as Iba1 and CD68 are effective, reflecting the involvement of immune responses in the central nervous system [150].

At the level of the lungs, fentanyl’s effects include pulmonary edema, respiratory depression, and alveolar damage. A specific marker like SP-A or SP-B can help assess alveolar integrity, which is often compromised in opioid-related deaths [151]. Aquaporin-1 can be used to evaluate fluid balance and detect the presence of pulmonary edema. Aspecific markers such as CD15 and MPO are useful for identifying neutrophilic infiltration and acute inflammation, while CD31 or VEGF can be employed to assess vascular damage and endothelial integrity [152,153].

In the heart, fentanyl has been associated with arrhythmias, QT prolongation, and myocardial fibrosis. Specific markers like cTnI are essential for detecting myocyte injury and necrosis, while desmin can evaluate the structural integrity of cardiomyocytes [154,155]. To identify fibrotic changes, collagen I and III can be used as markers of extracellular matrix deposition. Aspecific markers such as CD3, CD4/CD8, and vWF are valuable for identifying immune-mediated myocardial damage and endothelial dysfunction [156].

In the liver, fentanyl use can lead to hepatocyte necrosis, portal inflammation, and fibrosis. A specific marker such as HepPar-1 can highlight hepatocyte-specific cytoplasmic antigens, making it useful for assessing cellular damage [157,158]. Inflammatory responses can be evaluated using IL-6, while cleaved caspase-3 serves as a marker for apoptosis in hepatic cells. Aspecific markers like α-SMA can detect stellate cell activation and fibrosis, and HCcAg can be used to confirm hepatitis C infection, which is common in intravenous drug users [159,160].

In the gonads, chronic fentanyl use disrupts the hypothalamic–pituitary–gonadal axis, leading to hypogonadism and impaired germ cell maturation [123]. Specific markers like SOX9 are useful for evaluating Sertoli cell function, while inhibin-α highlights Leydig cell activity. To assess cellular proliferation within the germinal epithelium, Ki-67 is an effective marker [161].

By combining specific and aspecific markers, immunohistochemical investigations provide a detailed picture of fentanyl’s impact on organ systems, offering valuable insights for both clinical and forensic contexts.

### 5.6. Toxicological Investigation

Accurate toxicological analysis requires the proper collection and preservation of biological samples, including blood (preferably from the femoral vein), urine, vitreous humor, gastric contents, bile, and solid organ tissues such as the brain, lungs, liver, and kidneys. Hair samples from the nape or pubic region can provide a long-term history of drug exposure [136,162]. Additionally, fentanyl and its metabolites are often detected in encephalic tissues and vitreous humor, offering supplemental evidence in cases of overdose [163].

Illicitly manufactured fentanyl is frequently combined with other substances, complicating toxicological analysis and increasing the risk of fatal outcomes [164]. For instance:

- Alcohol and benzodiazepines: These central nervous system depressants exacerbate fentanyl-induced respiratory depression, increasing the likelihood of apnea and cardiac arrest [165].
- Cocaine: This stimulant can enhance cardiovascular effects, such as tachycardia and arrhythmias, which, when combined with fentanyl, can lead to sudden cardiac death [48].

Polydrug use also interferes with fentanyl’s metabolism. For example, ritonavir and calcium channel blockers, such as diltiazem, increase fentanyl plasma levels by inhibiting CYP3A4, while fentanyl itself can inhibit the clearance of sedatives like midazolam [166].

The rising prevalence of xylazine as an adulterant in fentanyl-related deaths has introduced significant challenges for toxicological investigations. Xylazine is increasingly co-involved in overdoses, often exacerbating the lethal effects of fentanyl [39]. A recent study analyzing xylazine-associated deaths in Michigan highlighted its growing prevalence in the illicit drug supply and underscored the complexities it adds to post-mortem toxicology testing. From October 2019 to June 2023, 100% of xylazine-positive deaths were found to also involve fentanyl, consistent with national trends of co-detection in drug-related fatalities [167].

However, its detection in post-mortem investigations remains inconsistent, hindering accurate assessments of its role in fatalities. One major issue is the variability in toxicological testing protocols across jurisdictions. Many laboratories do not routinely screen for xylazine, resulting in an underreporting of its presence in overdose cases [168]. Moreover, even in laboratories equipped to test for xylazine, variability in its post-mortem blood concentrations, which ranged from 5.2 to 200 µg/L in recent analyses, complicates the interpretation of its toxicological significance. This range is consistent with previous literature, yet no established thresholds exist to determine its toxicity levels in humans [167].

Even when xylazine is included in toxicology panels, its short half-life of approximately 25–30 min complicates its detection, particularly in cases with delayed sample collection. This transient presence in the bloodstream may lead to a misclassification of xylazine-related deaths as solely fentanyl-induced. Additionally, naloxone detection was notably lower in xylazine-positive cases (30%) compared to opioid-positive but xylazine-negative cases (21.2%), highlighting gaps in the use of naloxone in overdose scenarios involving xylazine. This finding underscores the critical need for increased naloxone distribution and public education about its limitations in addressing non-opioid components of polydrug overdoses [167].

### 5.7. Genetic Influences on Metabolism and Susceptibility

Understanding fentanyl’s pharmacokinetics and pharmacogenetics is essential in forensic investigations, particularly for interpreting post-mortem findings and determining the cause of death [72]. As previously described, due to its lipophilicity, fentanyl is quickly redistributed into fatty tissues after death, leading to variable blood concentrations that may not accurately reflect ante-mortem levels [169]. This redistribution complicates the differentiation between therapeutic use, misuse, and overdose. Ante-mortem doses cannot reliably be inferred solely from post-mortem blood concentrations [170].

As previously discussed, pharmacogenetics plays a significant role in fentanyl-related fatalities by influencing individual variability in metabolism and susceptibility to toxicity [171]. To determine the potential impact of various polymorphisms, the following key genes should be investigated during post-mortem examinations:

- *CYP3A4* and *CYP3A5*: These enzymes metabolize fentanyl into its inactive metabolite, norfentanyl. Reduced-function polymorphisms in these genes can lead to slower clearance, resulting in prolonged exposure and an increased risk of respiratory depression [172,173].
- *OPRM1*: This gene encodes the MOR, which mediates fentanyl’s central effects. The A118G polymorphism in *OPRM1* alters receptor binding affinity, with certain genotypes associated with enhanced sensitivity or tolerance to fentanyl [174,175].
- *ABCB1*: This gene encodes P-glycoprotein, which regulates fentanyl’s transport across the blood–brain barrier. Variants such as C3435T can influence fentanyl’s central nervous system concentrations, impacting both efficacy and toxicity [176,177].

Genetic testing for these polymorphisms can provide valuable insights into the mechanisms behind fatal cases, particularly when polydrug use or comorbidities are involved.

## 6. Discussion

Although fentanyl misuse has been documented since its discovery, its abuse has surged to alarming levels due to illegal trafficking and its replacement of traditional opioids like heroin [178]. In the United States, fentanyl-related fatalities have reached epidemic proportions, with approximately 70,000 deaths reported in 2022 alone [179]. Europe is also experiencing rising mortality rates; Sweden documented its first fentanyl-related deaths in 1997, and since 2012, the European Monitoring Centre for Drugs and Drug Addiction (EMCDDA) has reported growing availability and abuse of fentanyl and its analogs [180].

In Italy, fentanyl abuse is becoming an emergent concern. Between 2018 and 2023, law enforcement agencies seized 123.17 g of fentanyl powder, equivalent to thousands of lethal doses, alongside tablets and other formulations. Recognizing the severity of the issue, a law enacted on 28 July 2020 classified fentanyl analogs as illegal substances. Despite these measures, fentanyl continues to pose a national and international emergency [181]. The national Drugs Policies Department has outlined measures such as increasing police oversight of the precursors and chemical substances used in fentanyl production, enhancing web monitoring to prevent trafficking, and tightening controls on fentanyl distribution within hospital pharmacies [181]. However, despite these initiatives, significant challenges remain. Fentanyl’s high potency means that even small quantities can cause mass intoxications, straining public health systems. Moreover, the proliferation of fentanyl analogs, which often bypass existing regulations due to their chemical variability, complicates enforcement.

One factor driving the crisis is the accessibility of fentanyl via the dark web. Online platforms enable anonymous distribution of fentanyl and its analogs, complicating enforcement efforts. Furthermore, fentanyl’s lower cost, higher potency, and ease of adulteration with other substances make it an attractive option for both users and dealers [37,38]. This widespread substitution of fentanyl for heroin amplifies the risks, as users are often unaware of its presence, significantly increasing the likelihood of overdose.

For a fentanyl-related death to be accurately identified, a multifaceted forensic investigation is essential. This begins with a thorough judicial inspection, which often reveals the drug paraphernalia commonly associated with fentanyl abuse, such as syringes, needles, spoons, aluminum foil, or transdermal patches. A detailed external examination of the body can uncover telltale signs, including needle marks, purple discoloration indicative of respiratory depression, frothy material around the mouth (referred to as the “mushroom plume”), and pupillary constriction (miosis). Together, these findings lay the groundwork for an autopsy that, when combined with toxicological and histopathological analyses, provides crucial insights into fentanyl’s effects on key organs, including the brain, lungs, heart, liver, and gonads [129]. While the autopsy remains the gold standard for determining the cause of death in fentanyl-related cases, future studies should focus on deepening our understanding of fentanyl’s specific impact on organ systems. Histological and immunohistochemical investigations can provide more detailed insights into organ-specific damage, particularly in the brain, lungs, heart, and liver [182]. These studies could identify novel markers of fentanyl-induced toxicity, improving diagnostic accuracy and advancing forensic methodologies.

Additionally, the role of polydrug use in fentanyl-related deaths warrants further investigation. Understanding the pharmacokinetic and pharmacodynamic interactions between fentanyl and substances such as alcohol, benzodiazepines, and stimulants like cocaine is critical for assessing the compounded risks of polydrug abuse. Advanced toxicological techniques should be applied to quantify these interactions and establish their contribution to fatal outcomes [183]. Moreover, as previously described, the identification of xylazine remains challenging. Standardized toxicological protocols and increased awareness of xylazine’s role in drug-related deaths are critical. Expanding routine toxicology panels to include xylazine, improving post-mortem sampling techniques, and developing guidelines for interpreting xylazine levels are necessary steps to address this emerging issue [167,168].

Pharmacogenetics also holds significant potential for advancing forensic investigations. Studies focusing on genetic polymorphisms in key enzymes (e.g., *CYP3A4* and *CYP3A5*), transporters (e.g., *ABCB1*), and opioid receptors (e.g., *OPRM1*) could help explain individual variability in fentanyl metabolism, efficacy, and toxicity [72]. Future research could explore integrating genetic testing into standard forensic protocols, enabling personalized interpretations of post-mortem findings.

Finally, the impact of fentanyl analogs on organ damage and their pharmacological profiles remains underexplored. As these substances differ in potency and metabolism, comparative studies are essential for developing comprehensive forensic tools that can adapt to the evolving drug landscape. In this way, the use of artificial intelligence tools could be very helpful in the future [184].

Addressing the fentanyl epidemic requires not only robust enforcement and prevention strategies but also a concerted effort in scientific research to better understand its effects and enhance medico-legal practices. Additionally, enhancing collaboration between toxicologists, forensic pathologists, and public health officials can ensure more accurate documentation and an understanding of xylazine’s impact, ultimately improving responses to this growing public health crisis [39,47].

## 7. Conclusions

Fentanyl is a potent synthetic opioid widely used for its rapid onset and multiple routes of administration, making it an effective analgesic for chronic pain management and intraoperative anesthesia. However, its accessibility through illegal channels, low cost, and extreme potency have made it a major drug of abuse worldwide. Its misuse is associated with severe side effects, including central nervous system depression, respiratory depression, cardiac arrhythmias, hypotension, coma, and death.

Fentanyl trafficking and abuse have become escalating problems, especially as opioid-related deaths continue to rise globally. In the United States, programs such as the National Forensic Laboratory Information System (NFLIS) and the National Drug Early Warning System (NDEWS) monitor emerging drug trends. NDEWS also collaborates with the European Monitoring Centre for Drugs and Drug Addiction (EMCDDA) and Europol to exchange information and maintain an early warning system for new psychoactive substances. Despite these efforts, the measures implemented thus far have proven insufficient to curb the fentanyl epidemic. Additionally, the co-administration of xylazine presents distinct challenges for the scientific community.

To combat this crisis effectively, stricter controls must be implemented across all stages of fentanyl’s lifecycle, including production, medical distribution, and trafficking prevention. Enhanced regulations and international collaboration are crucial for reducing both the medical misuse and illicit circulation of fentanyl.

Given the increasing number of fentanyl-related deaths, particularly in the United States and Europe, it is imperative to establish standardized medico-legal protocols. These protocols should aim to improve the accuracy and efficiency of diagnosing fentanyl-related deaths, guiding forensic investigations in suspected overdose cases. Furthermore, integrating pharmacogenetic testing and toxicological analyses into routine forensic practice will enhance the identification of individual susceptibilities, providing valuable insights into the causes of fatal outcomes.

The growing prevalence of fentanyl misuse also underscores the importance of public health interventions, such as harm reduction programs, education on opioid risks, and improved access to addiction treatment. Strengthening these measures will not only help mitigate the impact of fentanyl but also address the broader opioid epidemic.

## Figures and Tables

**Table 1:** Fentanyl administration methods, effect time, and common users.

Administration Method	Effect Time	Duration	Frequent Users
Intravenous	1–2 min	2–4 h	Surgical patients
Transmucosal	10–15 min	2–4 h	Illicit users
Sublingual	5–10 min	Variable	Illicit users
Intranasal Spray	5–10 min	30 min	Chronic pain patients, illicit users
Transdermal Patch	Variable	8–16 h	Chronic pain patients, illicit users

**Table 2:** Toxicity and histopathology of target organs.

Organ	Toxicity	Histopathology
Brain	- Downregulation of opioid receptors; - Cognitive and emotional alterations (chronic); - Euphoria but also sedation (acute).	- Hypoxia in the hippocampus and cerebellum; - Neuronal apoptosis; - Microglia inflammation; - Altered distribution of microglia; - Vacuolization and gliosis of the affected regions.
Lung	- Hypercapnea; - Hypoxemia; - Chest wall rigidity; - Respiratory depression.	- Pulmonary edema; - Amorphous eosinophilic material in the alveolar spaces; - Intra-alveolar hemorrhage; - Inflammation of neutrophils; - Septic embolism.
Heart	- Hypocalcemia; - QT elongation arrhythmias; - Sudden cardiac death.	- Myocyte necrosis; - Endocarditis; - Fibrous connective tissue (myocardium).
Gastrointestinal system	- Nausea; - Vomit; - Constipation.	- Lymphocyte infiltration; - Portal inflammation; - Pyknosis and necrosis of hepatocytes.
Gonad	- Infertility; - Loss of libido and hypogonadism.	- Reduced maturation of germ cells; - Tubular diameter of the epithelium.

**Table 3:** Relevant immunohistochemical markers for each organ, the rationale, and the expected positivity.

Organ	Marker	Rationale	Expected Positivity
Brain	GFAP (Glial Fibrillary Acidic Protein)	Indicates astrocytic gliosis	+++
	NeuN	Assesses neuronal viability	++
	Cleaved Caspase-3	Marks neuronal apoptosis	+++
	Iba1	Reflects microglial activation	+++
	MBP (Myelin basic protein)	Evaluates white matter integrity	++
Lungs	Surfactant protein A (SP-A)/SP-B	Assesses alveolar integrity	++
	Aquaporin-1	Highlights fluid balance and edema	+++
	CD15	Detects neutrophilic infiltration	+++
	Myeloperoxidase (MPO)	Indicates acute inflammation	++
	CD31/VEGF (vascular endothelial growth factor)	Evaluates vascular endothelial damage	++
Heart	Cardiac troponin I (cTnI)	Identifies cardiac myocyte injury	++++
	Desmin	Assesses structural integrity of myocytes	+++
	Collagen I/III	Indicates myocardial fibrosis	++
	CD3/CD4/CD8	Reveals immune-mediated myocardial damage	++
	von Willebrand factor (vWF)	Highlights endothelial dysfunction	+++
Liver	Hepatocyte paraffin-1 (HepPar-1)	Identifies hepatocyte-specific cytoplasmic antigens	+++
	Cleaved Caspase-3	Marks hepatocyte apoptosis	+++
	Interleukin 6 (IL-6)	Reflects inflammatory response	+++
	α-Smooth muscle actin (α-SMA)	Indicates stellate cell activation and fibrosis	++
	Hepatitis C Virus Core Antigen (HCcAg)	Detects hepatitis C infection	++++
Gonads	SOX9	Highlights Sertoli cell function	++
	Inhibin-α	Marks Sertoli and Leydig cell activity	++
	Ki-67	Indicates proliferative activity	+++

Notes: Intensity definitions: (+): Weak positivity; localized faint staining. (++): Moderate positivity; visible across larger areas of tissue. (+++): Strong positivity; prominent staining indicative of significant protein expression. (++++): Very strong positivity; widespread, intense staining. Adjustments may be needed depending on the specific conditions of your experimental setup.

## Data Availability

All data are included in this manuscript.
